# Operationalising palliative care integration: Experiences of implementers in South Africa

**DOI:** 10.4102/phcfm.v18i1.5221

**Published:** 2026-03-30

**Authors:** Juanita O. Arendse, Virginia Zweigenthal, Liz Gwyther

**Affiliations:** 1 Department of Public Health, Faculty of Health Sciences, University of Cape Town, Cape Town, South Africa; 2 Western Cape Department of Health and Wellness, Cape Town, South Africa; 3 Department of Family, Community and Emergency Care, Faculty of Health Sciences, University of Cape Town, Cape Town, South Africa

**Keywords:** palliative care integration, implementation research, multidisciplinary team, community health workers, qualitative research, street-level bureaucrats, operational managers

## Abstract

**Background:**

South Africa’s 2017 National Policy Framework and Strategy for Palliative Care mandated the integration of palliative care (PC) into the public health system using existing resources.

**Aim:**

To explore PC implementers’: (1) experiences at least 1 year after implementation; (2) implementers’ capability in providing PC; (3) approaches to PC integration at selected sites and (4) perceived enablers and obstacles requiring managerial support.

**Setting:**

Healthcare facilities in the Cape Metro District (CMD) of South Africa.

**Methods:**

A qualitative, exploratory design was employed in five primary healthcare facilities classified as higher- or lower-morphine-usage sites based on dispensing trends. Purposive sampling identified 29 stakeholders: 22 implementers (clinical and support staff) and seven operations managers. Data were collected in four virtual focus group discussions and one in-depth interview with implementers and one in-depth interview with managers.

**Results:**

Three main themes emerged: (1) Provider capability: implementers reported variable confidence in clinical skills and in providing psychosocial support; (2) health systems response: participants cited insufficient staffing and time, limited training opportunities and inconsistent multidisciplinary team functionality and (3) provision of patient-centred PC: stakeholders emphasised the need for clear referral pathways, interprofessional communication and supportive leadership to ensure continuity of care.

**Conclusion:**

Palliative care implementers’ engagement in policy design and resource allocation is essential for sustainable implementation. Strengthening multidisciplinary teamwork, embedding PC content in undergraduate curricula and providing ongoing managerial support will enhance equitable access to quality palliative services.

**Contribution:**

Understanding how frontline stakeholders operationalise PC integration is essential for refining policy and practice in low-resource settings.

## Introduction

Understanding implementation is essential for policymakers and health system managers when introducing new interventions. Implementation research seeks to close the research–practice gap by assessing whether research impacts public health practice and whether evidence-based practice leads to quality improvement.^1^

Atun et al.’s^2^ framework provides a structured approach to examine how a new health intervention becomes embedded in a health system, considering the World Health Organization (WHO) health-system building blocks.^3^ According to this framework, the degree of integration depends on: (1) the problem addressed, (2) the complexity of the intervention, (3) adoption-system dynamics (including cultural norms and beliefs of stakeholders), (4) health-system characteristics, and (5) the broader contextual environment. This framework enables exploration of why integration varies across settings. The broader context of South Africa’s healthcare system is characterised by many challenges, primarily because of ‘lack of human resources, poor governance and management, and unequal distribution of resources’.^4^

Palliative care (PC) aims to ensure that patients with life-limiting illnesses do not experience avoidable suffering. By starting PC early, patients and families can better manage symptoms, prepare for the end of life and avoid needless hospital admissions and excessive use of health services.^5,6^ In 2017, South Africa’s National Policy Framework and Strategy for Palliative Care (NPFSPC)^7^ mandated that PC be integrated into the public healthcare system. In response, the Western Cape Department of Health and Wellness (WCDHW) launched a PC service using existing resources in 2018. Palliative care that is integrated into regular healthcare relies on functional multidisciplinary teams (MDTs).^8^ Multidisciplinary teams for PC can include doctors, nurses, allied health professionals, psychologists, registered counsellors, social workers, spiritual counsellors and volunteers, with access to physicians, other specialists and PC experts. An integrated team caring for the patient and caregiver enables a holistic approach to care, improves vigilance and addresses patients’ complex needs, particularly at the end of life.^6,9^ Multidisciplinary teams’ effectiveness also depends on communication between team members regarding patient and family caregiver care plans and support.^8,10^ Operations managers at the health facility level provide direct support and supervision to clinical and non-clinical teams, perform administrative duties and oversee the general management of facilities and hospital wards.^6^

Multidisciplinary teams are not widely established and maintained in South Africa, and embedding such teams into the health system requires time and effort, especially if PC is not integrated into undergraduate education, health system referral pathways, relevant policy and policymaker practices.^6,11^ Core multidisciplinary team (MDT) membership may vary between primary healthcare (PHC) facilities, given that allied health professionals are often shared among facilities,^8^ thus forming part of the extended professional network that also includes specialist support from hospices and higher levels of care.^11^

Community-based PC teams comprise community health workers (CHWs) and professional nurses employed by non-governmental organisations (NGOs), which are important for PC provision and are often contracted to support health services.^12^ The PC model is strengthened when community-based teams are included as part of the MDT, establishing a shared care model for patients through a community-oriented primary care (COPC) approach.^6,13^ The involvement of external parties is not without risk. DeMiglio and Williams^9^ note that facility teams in Canada often feel threatened by community-based PC service providers, and in the rural Western Cape, changes in the role of the CHW to focus on health promotion and disease prevention have resulted in role confusion.^14^

From a transdisciplinary perspective, nurses fulfil the role of operational managers at PHC facilities in South Africa, ensuring that their teams provide quality care to healthcare users and facilitate the implementation of new interventions. Gilson et al.^15^ conducted a study in the Cape Metro District (CMD), where the present study was conducted, and reported that nurse operational managers’ views and values influence health policy implementation and that they often feel excluded from policy decision-making. Further obstacles included work overload resulting in absenteeism, poor technical knowledge, limited time to ensure adherence to clinical guidelines, together with inadequate leadership, supervision and management skills, resulting in ineffective supervision at PHC facilities.^15,16^ Nursing staff shortages also contribute to poor delegation and limited time for supervision and administrative work.^17^

The CMD includes Cape Town in the Western Cape province, which forms part of the state-funded provincial health system, and comprises tertiary and central, secondary or regional, district and specialised hospitals and PHC facilities, including City Health municipal health services. Established stakeholder relationships exist between the provincial health system and NGOs, higher education institutions, City Health and the private healthcare sector.^6^

Frontline healthcare workers can be characterised as ‘street-level bureaucrats’ (SLBs), public sector workers who engage with the public and have significant discretion in performing their work.^18^ Loyens et al.^19^ refer to them as ‘street-level leaders’ – key influencers of practice, who ‘develop routines to avoid making endless individual choices’ in their complex work environment, using their discretion while considering the client and the organisation’s characteristics. Street-level bureaucrats have the discretion to make suggestions and decisions based on knowledge of the needs of those they serve, signifying the relevance of bottom-up theories of policy implementation.^20^

This study, conducted by the first author and supervised by the other authors as part of a doctorate,^6^ aimed to investigate the experiences of the implementing teams and operations managers to gain insight into their perspectives on factors influencing the integration of PC services at selected sites. Specifically, we wanted to determine the experience of PC implementers after 1 year of implementation, explore their capability in providing PC to patients with life-limiting conditions, understand the approaches to PC integration and identify their need for support from management in terms of enablers and obstacles.

## Research methods and design

### Design

A qualitative design was used to explore participants’ experiences of primary care facility-based PC implementation and integration into the healthcare system after 1 year or more at selected facilities in two sub-districts in the CMD.

### Setting and site selection

Because the indicator ‘number of patients accessing PC’ was not collected and ICD-10 code Z51.5 (‘encounter for PC’) was poorly captured, morphine dispensing was used as a proxy for PC service utilisation. Facility-level data for ‘morphine unspecified’ (presumed mist morphine typically prescribed at primary care level) and morphine sulphate (injectable; generally for inpatients or hospital-referred care) were obtained from the Provincial Health Data Centre (PHDC) for 2013–2018 (annual) and January 2019–June 2020 (quarterly), corresponding to the post-NPFSPC rollout period. Potential misclassification between categories could not be excluded; therefore, the datasets were combined to estimate total morphine utilisation, defined as medication prescribed and issued.^6^

Year-on-year proportional changes to 2019 (with projections for 2020 to stabilise small-volume trends) informed site classification. Facilities with an increase in morphine use of ≤ 15% were designated Low morphine usage site (LMUS); higher-usage facilities were designated Higher morphine usage site (HMUS). In sub-district one (SD1), all three facilities met LMUS criteria; in sub-district two (SD2), facilities exhibited an average increase of ≥ 38%. For broader context, morphine use across CMD PHC facilities and district hospitals showed an overall 3% decrease from 2019 (*n* = 5848) to 2021 (*n* = 5646); by sub-district, four decreased by 3% – 35% and four increased by 9% – 19%. Two-sample tests of proportions (z-tests) indicated that the sub-district increases were statistically significant (*p* < 0.05).

Site selection prioritised contrast in utilisation. As no LMUS facilities were identified in SD2, three HMUS sites were purposively selected there; in SD1, three LMUS sites and one HMUS site (a district hospital) were included. Sub-district one comprised two district hospitals and six community day centres; SD2 comprised one district hospital, one 24-h community health centre and two community day centres. To assess contextual influences, PC champions on the Palliative Care Task Team (PCTT) in both sub-districts were consulted regarding leadership transitions, operational changes and cross-facility staffing. Following this review, one SD1 facility was excluded (newly established; very low morphine dispensing), and one SD2 facility was excluded (shared clinical leadership with another site under the same family physician since 2015). Although two SD1 LMUS facilities reported to the same facility manager, they had distinct operational and clinical managers and were therefore retained. Final site labels – LMUS and HMUS – are reported in Table 1.

**TABLE 1:** Summary of study sites and participants.

Morphine usage of site	Site description	Morphine usage data			Implementers (FGDs)		Operations managers (one-on-one interviews)		Number of staff trained in PC at the facility
		code	Year	Usage (*n*)	Participants (*n*)	Occupation	Participants (*n*)	Occupation	
Higher	Site 1: CDC (8 hour facility)	HMUS1	2018 2020	43 66	5	family physician social worker pharmacist admin clerks (x2)	1	assistant manager in nursing	2
	Site 2: CHC (24 hour facility)	HMUS2	2018 2020	224 338	7	family physician doctor clinical nurse practitioners (x2) social worker pharmacist admin officer	2	operational managers in nursing	4
	Site 3: District Hospital	HMUS3	2018 2020	172 402	6	family physician social worker Allied health practitioners (x2) pharmacist admin officer	2	clinical manager family physician	5
Low	Site 1: CDC (8 hour facility)	LMUS1	2018 2020	85 92	3	doctor pharmacist admin clerk	1	assistant manager in nursing	1
	Site 2: CDC (8 hour facility)	LMUS2	2018 2020	60 30	1	doctor†	1	operational manager in nursing	0
**Total number of participants**	-	-	-	-	**22**	-	**7**	-	**12**

*Source:* Adapted from Arendse JO. An assessment of the integration of palliative care in the health system of the Cape Metro District of South Africa. University of Cape Town, Faculty of Health Sciences, Department of Public Health and Family Medicine; 2023. Available from: http://hdl.handle.net/11427/39266

PC, palliative care; CDC, community day centre; CHC, community health centre; FGD, focus group discussions.

† , FGD was converted to an interview.

### Participants

Two groups of facility participants were included: implementers and operations managers. Implementers were the clinical staff involved in providing PC services (doctors, nurses, pharmacy staff, social workers and allied health professionals) and non-clinical facility support staff and clinical staff not implementing PC (but who identify and refer potential patients within the health facilities). They met at least one of the following criteria: (1) assess patients for PC eligibility according to the Supportive and Palliative Care Indicator Tool (SPICT), (2) use referral documents and enrol patients in support groups, (3) convene support groups or provide telephonic support for PC patients and their family caregivers, or (4) draft PC care plans. In addition, facility support staff involved in patient folder management, personnel responsible for the dispensing of medication or clinical staff responsible for the identification and referral of patients to PC were included as implementers. The operations managers included nurse operational managers and facility managers responsible for the supervision of clinical and non-clinical teams, administrative duties and the general management of PHC facilities.^17^ In total, 29 participants were recruited: 22 implementers and seven operations managers.

### Sampling and inclusion

#### Data collection tool

A semi-structured interview guide^6^ (Online Appendix 1) with open-ended questions was designed to gain insights into implementers’ level of confidence in providing PC, identifying needs and access to ongoing care for patients and families. We sought to determine the influence of participants’ cultural values and faith orientation on providing PC; to understand the support from management; to identify enablers and barriers to the provision of PC; to ascertain referral processes to home and community-based care (HCBC) services; and to identify integration requirements for the future. Similarly, an interview guide was drafted^6^ (Online Appendix 2) for the virtual one-to-one in-depth interviews with the operations managers. This open-ended guide sought to determine their role in PC policy implementation, the support they offer to implementing teams and receive from management teams and the changes observed within the health system. The guide was further refined after the focus group discussions (FGDs) had provided more information that required further exploration and to enable triangulation of data.

#### Data collection

Four virtual FGDs and one interview took place at the five facilities via MS Teams (a video platform). For the planned FGD in LMUS2, five participants gave consent and confirmed that they would attend. However, only one participant arrived on the day at the designated time. This doctor wanted to proceed, and thus the FGD was converted into an interview with this one participant. After completion of the FGDs and one interview, one-to-one telephonic interviews took place with the operations managers for the selected sites. A research assistant (social worker) with FGD and research experience facilitated the FGDs and interviews.

#### Data management and confidentiality

The virtual FGDs and telephonic interviews were audio-recorded, and notes were taken by the research assistant and researcher. At the outset, the implementers were reminded that the proceedings were confidential. Names and images of participants were not captured. At the end of the sessions, debriefing took place to allow for note expansion and the generation of debriefing notes. The recordings were professionally transcribed and checked by the researcher before analysis.^6^

### Analysis

Inductive thematic analysis was used to explore the narrative emerging from the FGDs and interviews. The researcher became familiar with the data by repeated reading and immersion in search of meanings, patterns and key themes and concepts.^21^ An initial list of ideas was coded into meaningful groups using the NVivo software programme. The entire dataset was worked through to ensure equal attention to each data item. Extracted data included data that were coded many times, coded once and even uncoded, which were then further explored to identify subthemes and main themes. Subthemes and main themes were defined, named and captured in a codebook and in the software programme. A thematic map was also crafted and written up. Quotations that best illustrate the themes are provided in the text and referenced by type of focus group as indicated in Table 1.

### Trustworthiness and rigour

Credibility, transferability, dependability, confirmability and a reflexive approach were used to establish the rigour and trustworthiness of the reported findings.^6,22^ The first author, Juanita O. Arendse, worked closely with the study supervisors to identify pre-existing beliefs regarding possible enablers and obstacles, which were assessed at regular intervals. An intentional reflective process ensured that her assumptions and context were not embedded in the findings. All 12 transcripts were coded by the first author, and three were randomly selected and analysed by the study supervisors and an external party to ensure confirmability. All discrepancies were discussed and resolved by consensus. The study was conducted and reported in accordance with the Consolidated Criteria for Reporting Qualitative Research (COREQ) checklist.^23^

### Ethical considerations

Ethical approval was obtained from the University of Cape Town, Faculty of Health Sciences Human Research Ethics Committee (No. MREC REF: 058/2019). Beneficence and justice were considered throughout all interactions with staff, and attention was given to preventing harm and upholding non-maleficence. The rights of study participants were upheld, and attention was given to protecting their confidentiality and ensuring their anonymity and autonomy. Power differentials were considered, and issues of overt or subtle coercion were addressed by reiterating that participation was not compulsory and by recruiting an experienced research assistant to promote good practice and reduce discomfort, given that the first author is a senior manager in this region.

## Results

All healthcare worker categories in MDTs at the PHC facility level and district hospital level were represented in the three FGDs from the HMUSs. The years of work experience of the participants from the HMUSs ranged from less than 1 year to more than 20 years (mean 11.2 years, standard deviation [s.d.]: 6.69). Between five and seven informants participated in each FGD at the HMUSs. Participation ranged from one to three participants in the two FGDs from the LMUSs, and no nurses, allied health, or social work staff attended as a result of operational pressure. Informants’ work experience ranged from 11 years to more than 20 years (mean 12.75 years, s.d.: 1.5).

Seven operations managers participated. The managers ranged in age from 44 years old to 51 years old (mean 47 years, s.d.: 2.71); five were female. Their years of experience as operations managers varied, ranging from 3 years to 13 years (mean 7.5 years, s.d.: 3.73). Of the 29 participants, 19 were female and 10 were male.

Of the two LMUS facilities, one implementer had formal PC training compared with 11 implementers across the three HMUS facilities.

### Themes

**FIGURE 1:**
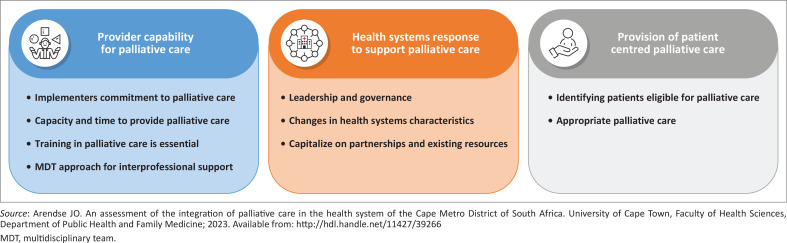
Map of the main themes and subthemes generated from the implementer and operations managers’ interviews.

Three main themes – *Provider capability for PC, Health systems response to support PC*, and *provision of patient-centred PC* – were generated from nine subthemes based on 36 codes. The subthemes and main themes are illustrated in Figure 1 and are discussed in turn in the subsections that follow.

#### Theme 1: Provider capability for palliative care

In all interviews, implementers and operations managers voiced their commitment to the provision of PC and highlighted the capacity, time and training required to provide PC services. The need to function as part of an MDT that could provide interprofessional support at the facility level was also highlighted.

**Subtheme: Implementers’ commitment:** Many informants’ personal experiences underlay their commitment to PC, including having lost a loved one with or without access to PC, having themselves required PC at some point or having a mentor who practised palliative medicine:

‘It was [*a*] personal journey having lost my dad with prostate cancer in 2014 … I put myself in the shoes of the family members … I know what I’m talking about because I have felt those feelings also.’ (HMUS 2, FGD, clinical nurse practitioner)

Informants raised concerns about adequate support for family caregivers who deal with end-of-life care and about the need for the health system to better respond to their needs. Some informants had not focused on the provision of PC and volunteered that they were willing to do more. Family physicians shared that their in-training rotation in PC had been invaluable in preparing them to offer PC. One family physician indicated that although PC was not part of their rotation, they requested a PC placement to become more knowledgeable in this field.

Policy negotiated at provincial and national levels hampered the initial buy-in from implementers, and more effort was then required to secure their commitment. The WCDHW needed to find a mechanism to ensure that the voice of the implementer is heard and included when developing policy or making changes to service delivery:

‘One of the obstacles that I found, that when the manager hands over the policy, and you haven’t been part of the team that put it together, or the staff in the facility was not involved … then the buy-in is very difficult.’ (HMUS 2, Interview, operational manager in nursing)

Informants maintained that once communities and staff became more aware of PC service provision, demand might increase. This would result in the need for more PC clinics at PHC facilities, or the weekly frequency of the PC clinics might need to be increased.

The operational managers believed that it was their responsibility to ensure the implementation of any new policy, but that the outcomes would depend on their context and resources:

‘I’m accountable to see that that policy is then fully implemented … And if it’s not … I must give the answers: this or that was the reason. But now it’s almost like it’s a new policy. It must be part of your services … to implement it is basically, it’s up to you.’ (LMUS 1, Interview, assistant manager in nursing)

**Subtheme: Capacity and time:** Palliative care requires time, staff and emotional capacity. Informants believed that current service pressures made it impossible to spend adequate time with patients and families. Implementers often needed to write prescriptions for morphine without seeing patients, which presented a barrier to engaging patients and families regarding their perceptions and use of morphine. Furthermore, these patients were often added at the end of a long and exhausting day, resulting in participants having to work overtime. Many implementers, particularly from the LMUSs, disclosed that they had burnout. Implementers in all FGDs insisted that additional PC-trained staff were needed to cope with the increasing demand:

‘We need to spend … time with the family and the patient … these scripts that just come to your room from assistants, they just quickly write up the morphine … that doesn’t work, you have to sit with a patient [*and*] find out what do they believe about morphine … you need to train them when to use it … And if you do it properly, there’s a good chance that it actually will work.’ (LMUS 1, FGD, pharmacist)

‘You will burn out if you do it on your own … Palliative care is not morphine only … Nobody can do [*it*] alone.’ (LMUS 1, FGD, doctor)

The implementers frequently develop long-term relationships with patients and members of the communities they serve, resulting in a significant emotional impact when these patients require PC or end-of-life care and ask difficult questions. The operations managers at some facilities voiced the importance of caring for the implementers. Many had an open-door management style and held regular group engagements for staff to talk freely in a confidential environment. All informants expressed commitment to providing PC services but required time and resources to provide quality care to all patients. They advocated for a plan that should include adequate human resources at the local level, with thorough consultation taking place with all stakeholders.

**Subtheme: Training is essential:** Training in PC was highlighted as essential for all role-players in the care pathway. This would facilitate the provision of quality care and equip staff to have difficult conversations with patients and caregivers. One rehabilitation professional was uncomfortable with providing PC and maintained that training would be needed to provide assurance that offering PC would be adequate care:

‘Our main … focus is always on a patient’s function and improving their level of functioning. … with palliative care, that often takes a backseat and then it’s … what make[*s*] the patient comfortable? Is that actually enough from a [*name of profession*] perspective? … From our perspective, … we could be doing more.’ (HMUS 3, FGD, allied health practitioner)

Implementers and operations managers maintained that although training in PC was essential and offered as several short courses (in person and online), it was difficult to release staff for training because of staff shortages, service pressures and staff discomfort with the provision of PC. Some participants added that when they applied to attend the PC short course, their applications did not progress beyond the People Development Unit at the sub-district office:

‘We need replacements from in the pharmacy when we go on training because the number of patients don’t decrease … when you are away.’ (HMUS 1, FGD, pharmacist)

Fortunately, the programme for family physicians at one university located in the Cape Metro included a rotation in PC, which meant that these family physician graduates were well positioned to lead the MDTs in PC and guide other staff members. Family physician informants indicated that they had selected PC as an elective because they understood the value of the training from other mentors in PC. Participants, particularly from HMUSs, expressed the need for PC training for all clinical and administrative personnel:

‘It would be beneficial if more people were trained in palliative care … you don’t have to be … a specialist to provide palliative care. And if we’re talking about integrating it into the system, then everybody from the clerk to the most senior doctor should … have some sort of training and know … how to approach and how to talk to patients and their family.’ (HMUS 2, FGD, family physician)

**Subtheme: Interprofessional support:** Informants described the importance of the MDT in supporting team members, improving patient-related communication and facilitating the provision of holistic care to the PC patient. Implementers trained in PC rely heavily on support from other team members who are qualified to provide care in areas where they feel ill-equipped. Specific mention was made of health professionals who offer rehabilitation services, social workers, dieticians and home-based carers (CHWs). They maintained that it would be helpful if PC experts located within the geographic care pathway could join their MDT meetings to provide guidance and insight. These could include PC experts from a local hospice or referring hospital or a family physician based at another facility.

Informants from several implementer groups were firm that communication between clinicians and pharmacy staff was essential to ensure that patients had adequate access to essential medicines. Titration of morphine was discussed, along with the need for pharmacists to provide additional morphine as required. Implementers were concerned about gaps in communication between referring hospitals within the care continuum, which then required patients to repeat their story again:

‘I’m very passionate about palliative care. And I like the fact that the patient and all the other disciplines are actually involved in the planning and in the management of the prognosis and the diagnosis. So … you will actually, as a team, be looking at one patient at a time in a holistic manner.’ (HMUS 1, FGD, family physician)

#### Theme 2: Health systems response to support palliative care

The health system should support PC through visible leadership, capitalise on existing resources and partnerships and create awareness of PC among communities and staff.

**Subtheme: Leadership and governance:** Implementers raised the need for on-site support from their area management team and the local health facility manager to guide them in implementing PC:

‘Our medical manager was very supportive of anything we wanted to do palliative care wise … like getting more pillows for patients … to looking at finding the finances and just being supportive rather than blocking any ideas …’ (HMUS 3, FGD, social worker)

Some implementer informants indicated that the sub-structure management team expected PC services to be rendered by simply providing the circular. However, what they needed from management was on-site support to guide the process and locate physical space for the service. They needed time to set out all processes and attend training. Some operations managers had not received any specific support from the management team other than the provision of the PC circular. Other implementers highlighted that their operations manager would coordinate the allocation of patients to ensure that time was available to see PC patients at the designated time of the weekly clinic:

‘We needed management to … allow one MO [*medical officer*] to be designated to palliative care for one afternoon. So, we came to a … compromise … where the [*MO*] would see twenty patients before 12 o’clock and then for the rest of that afternoon, her palliative care patients would come.’ (HMUS 2, FGD, clinical nurse practitioner)

The perspectives of operations managers on the actual implementation of PC services varied. Some viewed PC as the role of the hospice and realised that they would need to start taking ownership of it. Others required a firm instruction to include PC in the performance plans of staff, which made them accountable for service delivery, while a third group agreed that this was core business aligned with universal health coverage:

‘We’re so used to palliative care … being rendered via hospice, we still don’t see it as part of our service package. We weren’t given clear deadlines or … instruction … it was just here is the new policy, you need to streamline it into service delivery.’ (LMUS 1, Interview, assistant manager in nursing)

**Subtheme: Health system changes:** Some operations managers indicated that PC was not funded and that no additional funding was required, as the MDT members and other resources were available and should be appropriately utilised. On the other hand, others did not participate in any budgeting processes, and some described how they utilised savings in their facility budgets at mid-year to shift their budgets for PC and other pressing needs. Implementers raised concerns about poor access to specific equipment and consumables that PC patients needed for adequate home care, for example wheelchairs, bedpans and syringe drivers:

‘We almost always stay within budget. And we have a yearly capital expenditure meeting where all the departments come and engage. They tell us what they want. We see if we can get it, we prioritise … the budget is very flexible to enable clinical services and palliative care.’ (HMUS 3, Interview, clinical manager)

Demand generation is an important health system characteristic. Implementers and operations managers emphasised the value of creating awareness among communities and staff about PC and its availability; it should be communicated as part of a comprehensive package of care. They maintained that responsibility for this communication resided at all levels of the healthcare system. Creating awareness would help reduce stigma associated with PC, often regarded only as end-of-life care, which many people are not comfortable with. This information would encourage those in need of PC to come forward and express their needs:

‘The mindset of the personnel and the community needs to change that palliative care is not death care. It is an improvement of quality of life. But for them to believe that we need to actually deliver a service that can actually improve quality of life.’ (LMUS 1, FGD, admin clerk)

Informants articulated the importance of measuring progress in PC. Some argued that PC is not viewed as being as important as other priority services, such as HIV and TB treatment. Tuberculosis (TB) and HIV are tracked at facility level using tally sheets and are part of the performance indicator dataset included in the annual performance plan of the WCDHW, whereas PC was not. The WCDHW culture of measuring performance through outputs meant that simple measures for PC should be introduced and tracked at local level. These could include the proportion of clinical staff trained in PC at each facility; the number of functional MDTs that conduct folder audits, death reviews and track patient outcomes and the number of PC patients referred to CHWs for support:

‘Maternal health and child health and HIV and TB health – we’ve got very specific indicators, and we’ve got specific interventions and specific targets … the same should apply for palliative care because [*otherwise*] it is an area that is thought of as less critical.’ (LMUS 2, FGD, doctor)

The implementers described the value of having a geographic referral mechanism in place to facilitate the referral of PC patients. This would require accurate patient details to facilitate continued care.

**Subtheme: Utilise partnerships and existing resources:** The value of partnerships within geographic areas was emphasised. Participants mentioned NGO-operated hospices or intermediate care facilities (ICFs) and the CHWs who provide support to patients and their families. The ability to refer patients for dignified care was vital, and the process of referral was simple and embedded in the health system. Some operations managers remarked that they did nothing for PC patients other than refer them to the hospice or CHWs. In contrast, implementer groups from the LMUSs saw the opportunity to capitalise on the expertise within the hospice environment and discussed how they could engage this resource to provide support to PC services at the local facility level:

‘I need the people from hospice to come and help us. We need … help to teach the home-based care people how to work with morphine and with patients … we need people to help us and then we can help the other people around us.’ (LMUS 1, FGD, pharmacist)

Concerns were raised by implementers regarding the support that families had in the home, specifically when the patient reached the end-of-life stage. Community health workers were not trained in PC, which limited their knowledge regarding morphine use and the support they provided to the family at the end of life. The health system needed to reconsider its role and boundaries in providing adequate end-of-life support for patients and families:

‘The other thing that really worries me is end-of-life care … people need to go to the house to help there and you need to be able to support the family members and the patient … in that really difficult time. And we cannot leave the facility to do that, so palliative care will need another entity who will take over at the end stage.’ (LMUS 1, FGD, doctor)

#### Theme 3: Provision of patient-centred palliative care

Informants described what they regarded as important in the provision of patient-centred PC: identifying new PC patients and ensuring that existing patients are identifiable as they journey through the health system.

**Subtheme: Identifying eligible patients:** Implementers outlined their facility-specific identification mechanisms instituted to identify PC patients. Some facilities marked the patient card and folder of new or existing PC patients. To gain momentum in locating eligible PC patients within communities, some implementers indicated that they depended on CHWs to help identify and refer these patients to PHC facilities. Informants also described the current inadequate referrals from tertiary-level hospitals to PHC facilities for PC patients. Gaps in the information provided and challenges in locating the referring clinician at that level of care made the adequate assessment and management of down-referred patients very difficult for PHC clinicians:

‘We don’t have really clear communication from higher facilities like [*x*] and [*y*]. They just send the patient to us, so it’s like a discharge letter … No clear plan or no input from anywhere else like from their social worker, from their psychologist, from their dietician.’ (HMUS 2, FGD, social worker)

Some emphasised the benefit of having VULA, a widely available mobile phone referral application, to consult on and refer PC patients across the service platform. Standard care plans and stationery would also ensure continuity of care for PC patients:

‘Maybe have a set care-plan document, that all … team members can contribute to. So that we have … the same document for all palliative care. So we make sure that all the steps are managed adequately.’ (HMUS 3, FGD, social worker)

**Subtheme: Appropriate palliative care:** Palliative care provision should be relevant to the health system context and be sensitive to the patient and family’s needs. The language and culture of the patient and family were raised, along with the importance of including the family in decision-making. Support groups were seen as important. Ideally, allowing patients and families to take charge and set up their own groups would be appropriate. While adequate pain management was essential to ensure quality of life, and PC could never be appropriate or adequate without this, informants raised concerns about the possible misutilisation of morphine by some family members or carers, as well as the risk to children living in the home. They saw that there was no other option for managing morphine side effects and anticipated resistance from younger clinicians to prescribing morphine until pain was excruciating:

‘The family is refusing to give it to them, and withholding the morphine from them, thinking that by giving them the morphine, they will hasten their [*the patient’s*] death … Family members need buy-in … Morphine makes a lot of patients sick, more sick than they are, because there’s nausea.’ (HMUS 2, FGD, clinical nurse practitioner)

For many patients living in the district, the physical infrastructure was not conducive to end-of-life care, since families with children share these small spaces. The WCDHW should consider providing dignified rooms that allow families to remain with the patient during this period:

‘People in the community cannot afford [*carers*] to come to them and help them and they cannot bring the patients to the clinic … we need a place where people can die in peace with their family around them that’s not the emergency room at the hospital …’ (LMUS 1, FGD, doctor)

Differences were observed between implementers at the HMUS and LMUS in terms of support from the area (sub-structure) management teams and facility managers and stakeholder engagement. There were also differences in leadership and governance arrangements, knowledge about PC and functional MDTs. At the LMUSs, there was limited knowledge of PC policy, with greater dependence on partners, while the HMUSs’ implementers and operations managers had more insight into and understanding of PC.

## Discussion

Frontline service delivery is undertaken by nurses and clinicians who possess nuanced, context-specific knowledge of their operational environments. All interviews demonstrate that policy consultation and negotiation should include the opinions of the implementers – the SLB – during the initial policy development stage to ensure buy-in from these influential frontline public healthcare workers. Lack of SLB consultation, along with abuse of their discretionary powers, makes policies less likely to be implemented and requires more support at the frontline.^6,19^ Gilson and Lipsky^24^ elaborated that supervision is required to ensure adherence to standard operating procedures or policies. Our informants considered that policymakers lack insight into their context and are disconnected from their reality at the frontline, findings also noted by Davidovitz et al.^25^ in a study conducted in Israel and in a book by Lipsky.^18^

Given the demographic characteristics of the five facilities, the absence of PC training within the LMUS sites is consistent with their observed low levels of morphine utilisation. The contribution of nurses to PC is indispensable, and the absence of their perspectives constrains the depth and comprehensiveness of the data obtained from the LMUS facilities. This limitation was exacerbated by the absence of social workers’ and allied health workers’ participation in the LMUS FGD.

Palliative care training attendance necessitated absence from the workplace in contexts already constrained by staffing shortages, heightened service pressures and absenteeism, compounded by a coronavirus disease 2019 (COVID-19) pandemic-related backlog in routine PC training. Hofmeyr^26^ identifies insufficient prescriber confidence in the use of oral morphine within PHC services in the CMD and inadequate availability of oral morphine and palliative analgesia at primary care level.^27^

Interviews were conducted with implementers of policy at three higher (HMUS) and two lower (LMUS) implementing sites, identified by the volumes of morphine dispensed and their managers. Three themes emerged: provider capability for providing PC services, the health system’s response to support PC implementation and integration and the provision of patient-centred PC. These themes align with key elements for assessing integration in Atun et al.’s^2^ health system characteristics and adoption system. Our findings emphasise the critical role of the implementers and healthcare user needs as part of the adoption system, aspects that are considered but not emphasised by Atun et al. In particular, special attention must be given to capacity building within the planning and service delivery functions as part of the health system characteristics.

### Provider capability for palliative care

As noted by Morgan^28^ in research also undertaken in the CMD, experienced informants advocated that PC should be provided as part of the package of care. Their professional commitment to providing PC was shaped either by their own PC experience or by the influence of a PC mentor or training programme. This accords with Tehranineshat et al.,^29^ who reported that healthcare workers’ personal experience of suffering makes for compassionate care, which is core to providing PC. Similarly, McIlfatrick et al.^30^ found that the public’s interest in PC was shaped by their personal experiences or by those of a loved one needing PC.

The operational managers regarded leadership for the implementation of any new policy or intervention as their responsibility. However, they emphasised that the lack of consultation and clarity about implementation, support from the area management team, available resources and acceptance of PC by staff limits what they can deliver. This confirms what other researchers have found: implementer acceptance and leadership commitment to PC are essential for implementation and integration. Giannitrapani et al.^31^ and Stjernwärd et al.^32^ indicated that supportive leadership, along with interdisciplinary coordination and the identification of local champions, is essential to improve clinical commitment.

Our study revealed a culture of measuring performance through outputs and the inclusion of PC in HCW performance agreements. According to the WHO,^33^ ‘the inclusion of PC indicators in national health information management systems contributes to awareness raising and action on PC from health care managers and workers’. Goal three in the NPFSPC^7^ recommends the establishment of a system to monitor and evaluate PC programmes. However, proposed indicators have not yet been approved for inclusion in the national indicator dataset (NIDS) (Madikiana L 2022, personal communication, July 11), which will, in turn, determine the provincial indicator dataset.

Managers at the HMUSs regarded PC policy implementation as their core business and saw the alignment with components of universal health coverage. Although LMUS managers regarded PC as essential, some indicated that it was the responsibility of hospices, and others required an instruction to include PC as part of the service package and in staff performance plans. Palliative care provision requires adequate numbers of trained staff to cope with the patient load and sufficient time to ensure quality care.^34,35^ As PC is not routinely offered as part of undergraduate training for health professionals in South Africa, the time away from work to attend training was problematic in view of limited clinical personnel. The WHA resolution and the NPFSPC emphasise PC training as essential for all healthcare workers and providers,^7,36^ including CHWs who render care outside the health facility.^34^

The implementers depended on other team members to offer holistic care to patients and families. Multidisciplinary team members included staff who were PC trained and others not yet trained. Den Herder-van der Eerden,^11^ an authority on PC in Europe, advocated that an MDT approach facilitates interprofessional support, and the literature underscores the value of MDTs for PC.^8,35^ Multidisciplinary team development was considered essential by the study participants but would need to be developed in stages. The LMUS informants highlighted the value of MDTs but had not yet formed them, while MDTs at the HMUS were more developed. Fernando and Hughes^8^ note that these stages of development towards implementation commence with forming and then evolve through the stages of storming, norming and ultimately performing in the best interests of the patient and family caregivers.

### Design for palliative care implementation

Providing a policy circular via email is not adequate when communicating a new policy. The HMUSs’ implementers had access to unambiguous internal processes that provided direction on PC service delivery, and these processes were integrally supported by their operations managers. The LMUSs did not have such clear processes, and clinical service provision and care for patients were offered by one individual. As Hamdan et al.^37^ found, a well-consulted implementation plan is necessary, with standard-operating procedures that guide the implementation of PC as a new intervention.

Low levels of public awareness regarding PC are a challenge that hampers the development of a public health approach to PC, as found by McIlfatrick et al.^30^ Public awareness is needed to build public policy to support death, dying, grief and loss. Community awareness of PC reduces stigma, the misconception that PC is end-of-life care only and that this may increase patient demand for and access to PC,^38,39^ requiring co-ownership and work at all levels within the health system. Notably, increasing demand for PC could lead to a greater need for support for families of patients during the care journey and at the end of life and would need greater health system readiness to accommodate these needs. As informants remarked, public sector hospitals should consider providing access to end-of-life care beds for patients and accommodating family caregivers to ensure dignified deaths. In a post-bereavement survey^10^ conducted at two adult acute hospitals in Ireland, a case was made for end-of-life care beds at hospices. Furthermore, NGOs funded by the WCDHW could provide support for patients at home where possible.

Findings highlighted that capitalising on partnerships^40^ and using CHWs in the provision of home-based PC^9,13^ could greatly benefit PC support for patients and families, ultimately reducing unnecessary hospital admissions. Delgado-Guay et al.^41^ and Dumnui et al.^42^ found that PC support and good communication can reduce emergency unit admissions by 25%.

### Consideration for patient-centred palliative care

Findings suggest an urgent need for standardised referral mechanisms, care pathways, care plans and stationery to record patient care and management plans. Barriers to care were exacerbated by the inability to locate a clinician at the referring hospital and the unavailability of electronic patient records across the health service platform.^43^ All agreed that structuring MDTs at PHC facility or geographic-area levels could facilitate communication and engagement to address challenges and empower patients with the agency necessary for their care decision-making.^44^

Crowded housing conditions of many public sector-dependent patients are not conducive to accommodating end-of-life needs. Consequently, due consideration should be given to the provision of and access to end-of-life care beds at acute hospitals for patients and their family caregivers to ensure an environment conducive to a dignified death.^10^ An exploratory review of international PC policy in five high-income countries (Switzerland, England, Singapore, Australia and Ireland)^45^ found that hospitals do not make provision for end-of-life care, while patients and families report a preference for hospital admission in these settings. Donnelly^10^ reported that dying in an acute hospital was a common occurrence in developed countries, and the literature makes a case for end-of-life care beds at hospices. However, there is a continued preference in African countries for home care with support from the community.^45^

### Limitations

This study took place early in the PC policy implementation journey in one district in South Africa, so the eventual PC service delivery system may differ from its initial form and design. Nonetheless, lessons learnt from the unevenness in service delivery at an early-stage point to factors that need to be considered in policy implementation.^6^ Some facilities experienced service pressures on the day of the group discussions, which impacted negatively on the number of attendees at the FGDs. Other informants could not be released to participate; this resulted in one group having only one participant and another having three participants. Fewer LMUSs than HMUSs were included in the study because there were mostly HMUSs in the two sub-districts. This imbalance may have skewed the findings towards the views of the HMUS implementers and operational managers.

## Conclusion

Frontline implementers need leadership continuity that is visible and supportive, protected time, basic PC training for all cadres, including administrative staff, a structure for MDTs, a standard functional referral system for all patients and care plan tools. Immediate managers can guide changes in service design, patient access and flow that lead to quality PC services and linkage to hospices and HCBC. Monitoring and evaluation, reporting and documentation for PC should be standardised. Palliative care training should be included in undergraduate training for all health professionals in South Africa and encouraged and supported by academia and health system leaders. Implementers are key contributors to policy and should be included in policymaking from its inception to facilitate consideration of context, resource needs and allocation and ultimately policy buy-in.
